# Supplementary material for: Identification of photoperiod-induced specific miRNAs in the adrenal glands of Sunite sheep (Ovis aries)
Source: Front Vet Sci. 2022 Jul 22;9:888207. doi: 10.3389/fvets.2022.888207 (PMC9354845; doi:10.3389/fvets.2022.888207)
Supplement: Supplementary file 1 [file Data_Sheet_1.ZIP › Supplementary file/miRDeep2 file.zip!/miRDeep2 file/Know_Structure/SPLP42A2/oar-mir-221.pdf]

miRBase precursor : oar-mir-221
Total read count : 4651
oar-miR-221 read count : 4224
remaining reads : 427

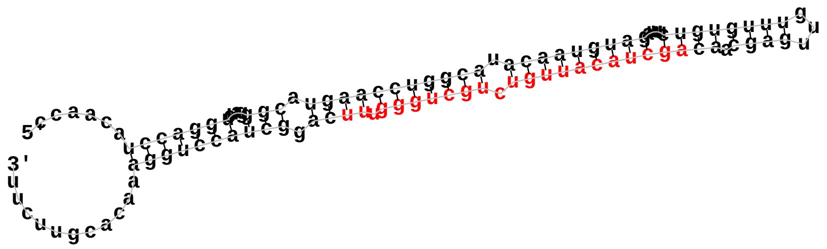

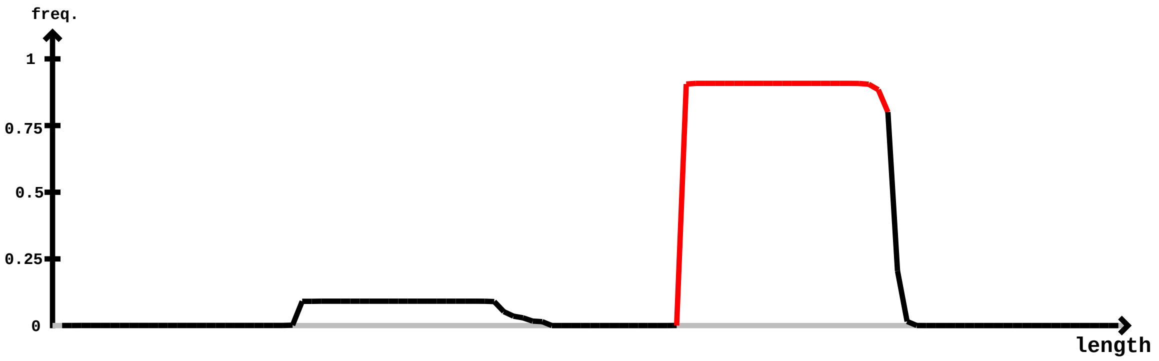

oar-miR-221

```
5'- ccaacauccaggucuagggcaugaaccuggcauacaauguagauuucuguguuuguugagcaacagcuacauugucugcuggguuucaggcuaccuggaaacacguucuu   -3'    exp
    ......((((((....(((.((((((((((.((((((((....((((((((...)))).))))))))))))).))))))).)))).))))))))))..........   reads      mm         sample
    ccaacauccaggucuagggcauga......................................................................................   1          0          seq
    .caacauccaggucuagggcauga......................................................................................   1          0          seq
    ......................aaccuggcauacaauguagau...................................................................   2          0          seq
    ......................aaccuggcauacaauguagauu..................................................................   2          0          seq
    ......................aaccuggcauacaauguagauuucugu.............................................................   1          0          seq
    .......................accuggcauacaauguaga....................................................................   1          0          seq
    .......................accuggcauacaauguagau...................................................................   4          0          seq
    .......................accuggcauacaauguagauu..................................................................   172        0          seq
    .......................accuggcauacaauguagauuu.................................................................   79         0          seq
    .......................accuggcauacaauguagauuuc................................................................   29         0          seq
    .......................accuggcauacaauguagauuucu...............................................................   56         0          seq
    .......................accuggcauacaauguagauuucug..............................................................   10         0          seq
    .......................accuggcauacaauguagauuucugu.............................................................   67         0          seq
    .........................cuggcauacaauguagauu..................................................................   1          0          seq
    .........................cuggcauacaauguagauuucu...............................................................   1          0          seq
    ...............................................................cagcuacauugucugcuggguuu.......................   1          0          seq
    ...............................................................cagcuacauugucugcuggguuuc......................   1          0          seq
    ................................................................agcuacauugucugcugg............................   2          0          seq
    ................................................................agcuacauugucugcuggg...........................   13         0          seq
    ................................................................agcuacauugucugcugggu..........................   96         0          seq
    ................................................................agcuacauugucugcuggguu.........................   391        0          seq
    ................................................................agcuacauugucugcuggguuu........................   2758       0          seq
    ................................................................agcuacauugucugcuggguuuc.......................   878        0          seq
    ................................................................agcuacauugucugcuggguuuca......................   71         0          seq
    ................................................................agcuacauugucugcuggguuucag.....................   1          0          seq
    .................................................................gcuacauugucugcuggguu.........................   9          0          seq
    .................................................................gcuacauugucugcuggguuuc.......................   3          0          seq
```
